# Low Light Conditions Alter Genome-Wide Profiles of Circular RNAs in Rice Grains during Grain Filling

**DOI:** 10.3390/plants11091272

**Published:** 2022-05-09

**Authors:** Hong Chen, Tao Wang, Zhiyou Gong, Hui Lu, Yong Chen, Fei Deng, Wanjun Ren

**Affiliations:** 1 Key Laboratory of Crop Ecophysiology and Farming System in Southwest China of Ministry of Agriculture, Crop Ecophysiology and Cultivation Key Laboratory of Sichuan Province, Sichuan Agricultural University, Chengdu 621000, China; chenhong_88@aliyun.com (H.C.); taowang_sicau@126.com (T.W.); zhiyou@stu.sicau.edu.cn (Z.G.); luhui@stu.sicau.edu.cn (H.L.); yongchen@sicau.edu.cn (Y.C.); ddf273634096@126.com (F.D.); 2 College of Life Science and Engineering, Southwest University of Science and Technology, Mianyang 621010, China

**Keywords:** rice, circular RNA, light

## Abstract

In animals and plants, circRNAs regulate gene expression and act as sponges that inhibit the activity of microRNAs. This study aimed to determine how specific circRNAs are expressed in rice grains at different stages of grain filling, under normal and low light conditions. We extracted total RNA from rice grains under low and sufficient light conditions. Deep sequencing was performed using circRNA libraries, and bioinformatics tools were used to identify the circRNAs. In addition, we analyzed targeted messenger RNA functions using two databases to predict the processes involved in rice grain development, and we conducted real-time PCR on 15 of the circRNAs as well as Sanger sequencing. During the grain development process, 8015 candidate circRNAs were isolated, among which the number of known circRNAs was 1661. We also found that the number of circRNAs changed with the time of development. Among them, six circRNAs acted as sponges that targeted more than two microRNAs at different stages of development, and these circRNAs showed a regulatory pattern consistent with the transcriptome sequencing results. More circRNA diversity was found under low light treatment compared to normal light. These findings reveal a possible link between circRNA regulation and the expression of the functional genes associated with photosignal-mediated rice grain development.

## 1. Introduction

Circular RNAs (circRNAs) are a class of noncoding RNA that are highly conserved and not easily degraded. In the 1970s, Sanger et al. [1] first discovered closed circRNA molecules in plant viruses. After the first discovery of circRNA in the roots of *Arabidopsis thaliana* (Linnaeus) Heynhold in 2014, multiple species of circRNA have been identified [2,3,4,5]. Several studies have shown that plant circRNAs have the potential to be miRNA sponges [2]. The primary function of circRNA is to regulate microRNA (miRNA) using the sponge mechanism, and it also regulates variable shearing and transmits signals over long distances [6]. Analysis of the circRNA–miRNA–mRNA regulatory network has shown that circRNAs might be involved in plant hormone signal transduction and porphyrin and chlorophyll metabolism during leaf senescence [6]. The sponge action of circRNAs shows that 6 out of the 62 circRNAs have miRNA-binding sites (three to eight sites) that can potentially regulate 26 distinct wheat miRNAs [4]. In cold-treated tomatoes, 102 circRNAs have the potential to act as miRNA sponges based on the predicted miRNA-binding sites for 24 distinct mature miRNAs [7]. CircRNAs play a key role during anther development, according to the circRNA–miRNA–mRNA network, which is involved in the anther development of *Brassia campestris* [8].

CircRNAs of various plants during growth stages have specific expressions in space and time. During the lifespan of *Arabidopsis* leaves, circRNAs express differentially at the growth-to-maturation stage of 4 days and the maturation-to-senescence stage of 16 days [6]. The species and expression of circRNA differed in the leaves and grains of rice [9]. However, there are few reports on the developmental stages in the important reproductive period. Previous reports have shown that circRNAs are expressed specifically under abiotic stresses, such as phosphate, chilling, and drought stress [2,4,7]. The expression level of 6012 circRNAs in *Arabidopsis* leaves were investigated and differed according to the light conditions and treatment times [2].

Light intensity is a key factor affecting plant growth. During the grain-filling period in rice, low light intensity affects the synthesis and transportation of photosynthetic products [10,11], such as sucrose, which is the main photosynthetic product in plants, the primary raw material for starch synthesis in grains of rice and other grain crops, and the most essential transport form in plants. Such effects of low light intensity influence the yield, seed setting rate, 1000-grain weight, and other yield components of rice [12,13]. Climate change has increased the frequency and intensity of rainy days with low light in Sichuan Province, which directly affects the growth and development of rice and other crops produced in the province [14,15]. The expression of circRNA under different developmental stages in rice under low light has not yet been reported.

With the increasing demand for high-quality rice, there is an urgent need to understand the molecular characteristics of the deterioration of rice quality under low light conditions and to provide a theoretical basis for rice breeding. In this study, 8015 candidate circRNAs were isolated and 6 circRNAs in different development were predicted to interact with miRNAs as miRNA sponges through target mimicry. The results indicate that the types of circRNAs in grains respond to light signals, and they are more likely to act on the filling process. These results will also help learn the roles of circRNAs in rice.

## 2. Results

### 2.1. Sucrose Content at Different Growth Stages under Different Light Conditions

**Figure 1:**
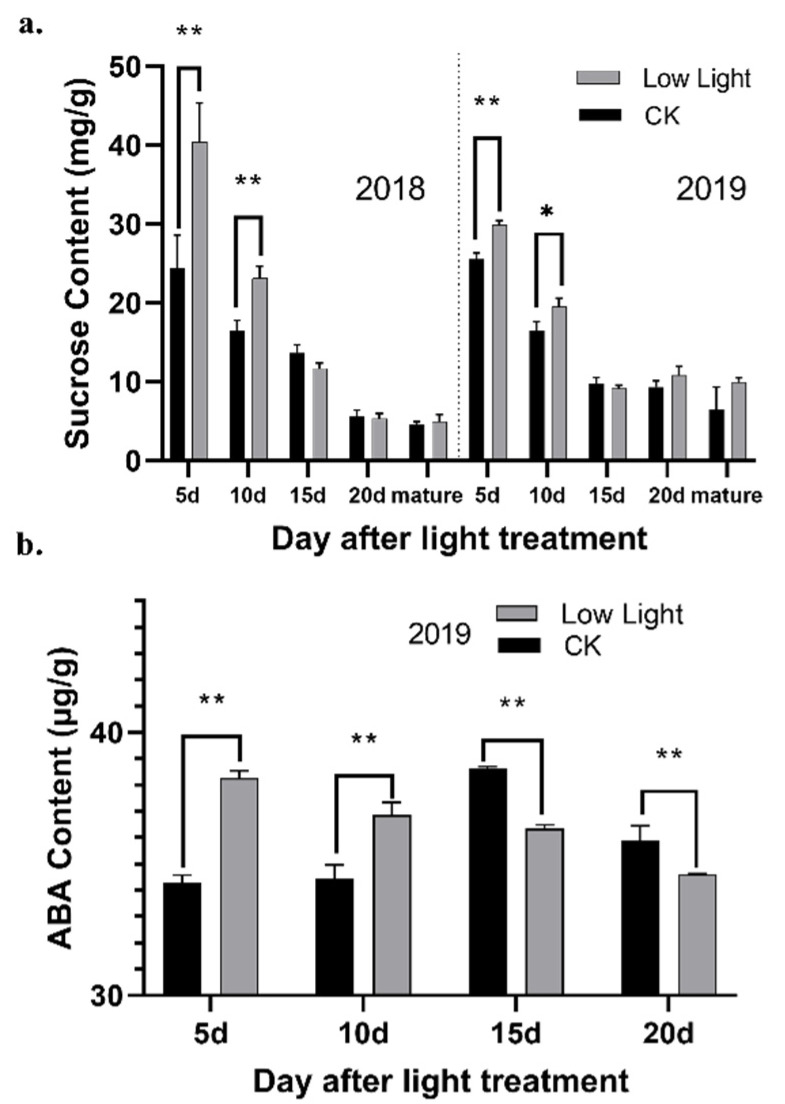
(**a**) Sucrose content in rice grains at 5d, 10d, 15d, 20d and the mature stage after light treatment in 2018 and 2019; (**b**) contents of ABA in rice grains at 5d, 10d, 15d, and 20d after light treatment in 2019. CK: normal light. * and ** indicates significance at *p* ≤ 0.05 and *p* ≤ 0.01.

The synthesis and transportation of sucrose were affected to some extent under low light conditions. The sucrose content in the grains gradually decreased during the grain development process. Under low light conditions, the content of sucrose in the rice grains was significantly higher than the control before 10 d (Figure 1a). This result might indicate that, under low light treatment, the synthesis and transportation of sucrose, the primary photosynthetic product in plants, follows a dynamic process.

Abscisic acid plays an essential role in the response of plants to various environmental stresses. In the developing grains in the control group, the ABA content first increased, then it reached a peak at 15 days, before gradually decreasing (Figure 1b). After the shading treatment, the ABA concentration in the grain was significantly higher than the control at the beginning, then it gradually decreased with time, before being lower than the control after 15 days. These findings are similar to other research that found that the ABA concentration in superior and inferior rice grains showed a decreasing trend during grain filling, but the ABA concentration decreased faster in the superior grains than in the weak grains at the same grain-filling stage [16,17].

### 2.2. Overview of the Circular RNAs in Rice Grains

**Figure 2:**
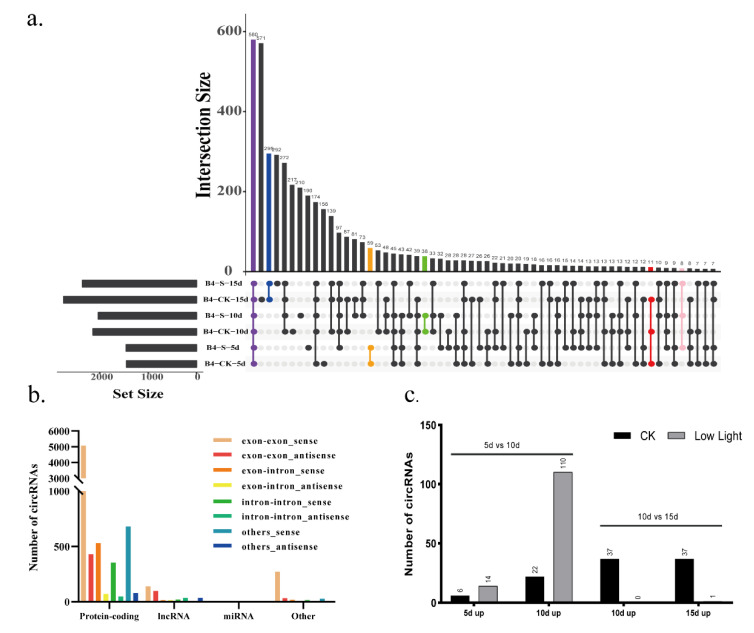
(**a**) The number of circRNAs shared between different light treatments or different grain-filling days: all samples (purple), the treatment and control after 5 days (orange), treatment and control after 10 days (green), 5, 10 and 15 days of control (red), and 5, 10, and 15 days of low light (pink). (**b**) The sequences of all circRNAs constitute a specific source. (**c**) The numbers of differentially expressed circRNAs were compared in the control at 5 and 10 days, and at 10 and 15 days after low light treatment. 5d up: the number of circRNAs expressed in grains after 5 days of low light was higher than that after 10 days of low light; 10d up: the number of circRNAs expressed in grains after 10 days of low light was higher than that after 5 or 15 days of low light; 15d up: the number of circRNAs expressed in grains after 15 days of low light was higher than that after 10 days of low light; CK: normal light; S-5d: low light for 5 days; S-10d: low light for 10 days; S-15d: low light for 15 days; CK-5d: normal light for 5 days; CK-10d: normal light for 10 days; CK-15d: normal light for 15 days.

There have been some reports on circRNAs in rice [2,4,7,18]. Of 30 experimentally validated circRNAs, 16 could be detected in both the leaf and panicle with nearly equal expression, 5 were more highly expressed in leaf, 2 were more highly expressed in the panicle, 3 were panicle-specific, and another 4 were leaf-specific [9]. However, the research on changes in circRNAs during the development of rice grains under different light treatments is limited. Circular RNAs are characterized by the occurrence of 3′-5′ connections in splicing reactions of individual RNA molecules. Based on the sequence reads, a total of 8015 circRNA candidates were identified by the Starchip, CIRCexplorer2, CIRI2, and CircRNA_finder software from a total of 18 samples from the two treatments of rice grains (Figure 2a). This is 240.48% more than that previously found in rice [9]. After filtering and comparing the data from the two databases, Plantcircbase and Plantcircnet, 1661 known circRNAs were obtained (Supplemental Table S1). A total of 7270 of the sources of circRNA formation were concentrated in coding RNA, with 5507 in the exon region, making up 85.73% of all sources in the exon region, 373 in other regions, with 306 in the exon region making up 4.76% of all exon regions, 367 from lincRNA, with 238 in the exon region making up 3.70% of all exon regions, and only 5 from miRNA from other regions (Figure 2b).

### 2.3. Identification of Differentially Expressed Circular RNAs

At different developmental stages, the number and expression of circRNAs were different. As fertility progressed, the amount of circRNA increased gradually under the control and low light conditions (Figure 2a). Overall, there was a small difference between the quantities detected under low light conditions (1455, 2032, 2360) and the control (1448, 2141, 2748) after 5, 10, and 15 days, respectively. There were 582 circRNAs detected at different filling stages and under different light treatments, accounting for 7.26% of the total number of circRNAs. This result shows that a certain number of circRNAs show stable expression during the grain development process regardless of the environment, and this plays an important role in grain-filling development (Figure 2a).

Different types of circRNAs play different roles during grain-filling stages, and the number of circRNAs involved at different stages varies greatly (Table 1). For example, 59 were detected in both the control and low light treatment after 5 days of low light, 38 after 10 days, and 295 after 15 days. A total of 43 circRNAs were detected at both 10 days and 5 days, and 272 were detected at both 10 days and 15 days. However, under normal light conditions, only 11 circRNAs were detected after each time period (Figure 2a). The number of upregulated expressions of circRNA at 10 days compared with 5 days under low light treatment was 110, which is significantly higher than that of the 22 in the control group (Figure 2c, Supplementary Table S3). The number of expressions circRNA regulated at 10 days compared to 15 days was mainly displayed in the rice grain under normal light.

The numbers in the figure represent the number of circRNAs with significantly different expression between different treatments—CK: normal light; S-5d: low light for 5 days; S-10d: low light for 10 days; S-15d: low light for 15 days; CK-5d: normal light for 5 days; CK-10d: normal light for 10 days; CK-15d: normal light for 15 days.

Under normal light, there were 28 differentially expressed circRNA on days 5 and 10, and 74 on days 10 and 15. When the light decreased, more differential expression occurred between 5 and 10 days under low light. However, there was no significant difference in the expression of circRNA between the two light treatments at any stage of grain filling. In grains, this may be due to the great diversity of circRNA, which affects the downstream expression, and makes the grain development different.

### 2.4. Putative Functions of the Regulations of Rice circRNAs Acting as miRNA Sponges

**Figure 3:**
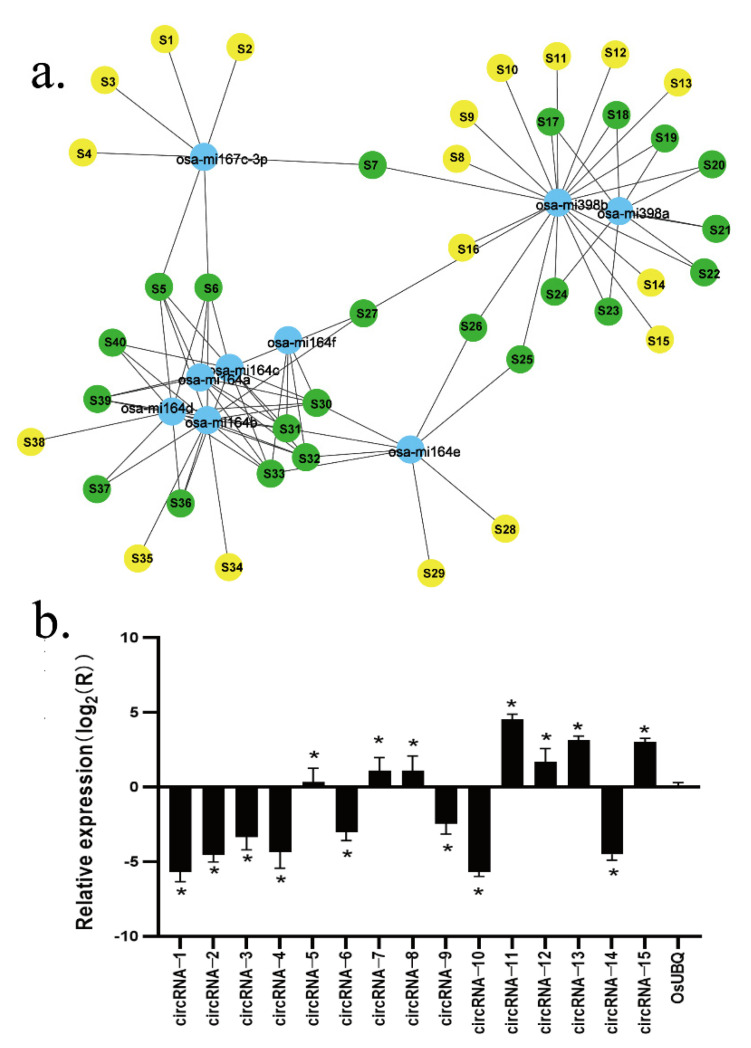
(**a**) Networked view of the differentially expressed circRNAs targeting miRNAs. Blue represents miRNA, green represents circRNA that targets more than two miRNAs, and yellow represents less than two miRNAs. (**b**) Validation of the differentially expressed circRNAs by real-time PCR assay and Sanger sequencing. See Table 1 for the circRNA names. Means of samples denoted with the “*” are significantly different at *p* < 0.05 according to the t-test compared to the control after 5 d. The ID represented by S1–S40 is in the Supplementary Table S7.

The RNAhybrid software predicted 612,874 targeted miRNAs, while TargetFinder predicted 3527, and 1398 were predicted by both prediction software programs, making the combined number of predicted targeted miRNA 615003. The six circRNAs (circRNA-7, circRNA-8, circRNA-11, circRNA-12, circRNA-13, and circRNA-15) predicted to be miRNA sponges showed positive amplification from the expected corresponding circular template, while the six circRNAs showed expression differences consistent with the transcriptome sequencing results. In addition, of the 15 circRNA candidate genes that were tested, 13 (except circRNA-5 and circRNA-15) showed expression patterns consistent with the RNA sequence results (Figure 3b and Supplementary Table S1).

Of the circRNAs that showed differences in light processing and development, 40 circRNAs have been reported as having known miRNA targets, including miRNA164, miRNA398, miRNA167, and various isomers (Figure 3a).

### 2.5. Functional Categorization of Predicted mRNAs

The differences in the biological processes in the control group from 5–10 days were mainly concentrated in carbohydrate metabolic processes (GO: 0005975), embryo development (GO: 00097990), lipid transport (GO: 0006869), and microtubule-based movement (GO: 0007018). These are essential metabolic and biological development processes in the development of grain embryos and endosperms. In terms of cell structure, monolayer-surrounded lipid storage bodies (GO: 0012511), cell walls (GO: 0005618), membranes (GO: 0016020), and extracellular regions (GO: 0005576) were identified. In terms of molecular function, 698 GO pathways were found to be involved. These were mainly related to hydrolase activity (GO: 000453, GO: 0016788), enzyme inhibitor activity (GO: 0004857), ion channel inhibitor activity (GO: 0008200), lipid binding (GO: 0008289), and carbohydrate metabolism and transport. The enrichment analysis that was conducted using the KEGG database showed significant differences in the expression of starch and sucrose metabolism (ma00500), phenylpropanoid biosynthesis (map00940), and glycerolipid metabolism (map00561). Analyses using the Wikipathway database found differences in seed development (WP2199), photosynthetic carbon reduction (WP1461), abscisic acid biosynthesis (WP626), sucrose metabolism (WP2623), and the ethylene signaling pathway (WP2851).

The difference between 10–15 days and 5–10 days after flowering in the control group was differences in electron carrier activity (GO: 0009055), protein heterodimerization activity (GO: 0046982), and the fructose 6-phosphate metabolic process (GP: 0005975). The main difference in enrichment found from the KEGG database was related to carbon metabolism (map01200), whereas the Wikipathway enrichment analysis found changes in photosynthetic carbon reduction (mapWP1461), glycolysis (mapWP2862), and the tricarboxylic acid cycle (mapWP2624).

When comparing the low light treatment with the control group at 5–10 days after flowering, there was specific differential expression of biological processes such as the abiotic stress response and photosynthesis, and when looking at cell structure, there were differences in the nucleosomes (GO: 0000786) and cell wall structure development (GO: 0005618). The expression of the cell wall invertase gene decreases during the development of vulnerable granules with multiple consequences: inhibiting the development of vulnerable granules, delaying the sucrose conversion rate and synthesis rate of vulnerable granules, forming a stagnant period of filling of vulnerable granules, and playing a role in the unloading of assimilating effects [19]. Under low light conditions, the grains of rice also showed similar weaknesses in development, which were similar to grout lag. The photosystem I reaction center (GO: 0009538, 7/2) also showed differential expression. Previous studies have shown that during rice grain filling, some photosynthesis still occurs in the grain. However, under low light conditions, photosynthetic products, such as sucrose, are reduced in the photosynthetically active tissues and thus the supply of these products to the grains is affected. Some photosynthetic cells in the rice grains appeared to be more active to make up for the inadequate synthesis of starch. In terms of molecular functions, protein heterodimerization activity (GO: 0046982), serine type endopeptidase inhibitor activity (GO: 0004867), and peroxiredoxin activity, which play important roles in the accumulation of nutrients, showed differences in activity. Compared with the control at 5–10 days after flowering, the enrichment analysis conducted using the KEGG database showed differential expression of photosynthesis antenna-proteins (map00196), flavonoid biosynthesis (map00941), and starch and sucrose metabolism (map00500).

In the low light treatment for the period 5–10 days after flowering, analysis of the Wikipathway database compared with the control group mainly showed changes related to sucrose metabolism (mapWP2623), ABA synthesis (mapWP626), and the chloroplast electron transport chain (mapWP2861). The content of endogenous ABA in grains under low light in this study was significantly higher than that of the control group at 5 and 10 days after flowering.

## 3. Discussion

There was more differential expression between leaves and roots [2,9], which express carbon and nitrogen metabolism during plant development, such as photosynthesis and nutrients. However, we only selected grains in rice, which are mainly concerned with the development and accumulation of embryos and endosperm. Moreover, light signal mainly affects grain development through the diversity of circRNA. For example, there were 571 circRNAs in the grains under normal light for 15 days and 292 circRNAs in the grains under low light for 15 days. These differences may be the cause of different grain development.

**Figure 4:**
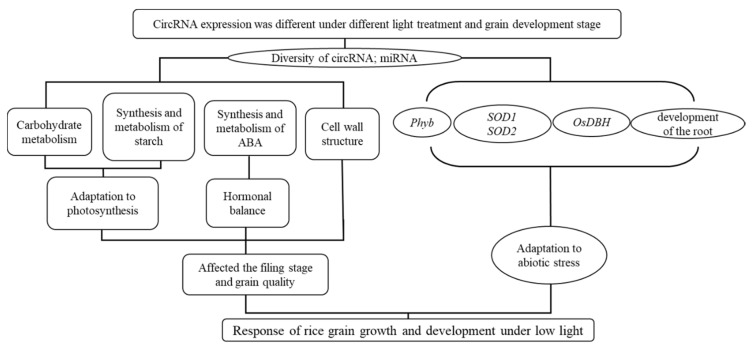
Possible regulatory mechanism involving differentially expressed circRNAs and their target genes in rice grains under weak light.

Meanwhile, we also speculated that circRNA’s influence on the development of rice grains through miRNA was mainly through hormone synthesis factors, helicase and sod-related enzymes, and ABA metabolism, before affecting the synthesis and metabolism of sucrose decomposable starch, and finally affecting the filling stage and grain quality (Figure 4). Meanwhile, miRNA was also used to respond to changes in external light stress (low light).

MiRNA is an important regulator of plant growth. At present, there are few studies on the mechanism of circRNA–miRNA sponges in plants. Liu et al. [20] found that pei-miR160a negatively regulated the expression of six PeARFs, five lncRNAs, and one circRNA by overexpressing pei-miR160a in the development of the root and shoot meristems of Populus.

The targeted miRNAs (miRNA164 and miRNA167) can influence the formation of root caps, lateral root development, and adventitious root development through auxin response factors [21,22], and they can also be induced by light conditions and participate in phyb-mediated signaling pathways [23]. In our study, their expression levels were also different at different grain-filling stages (Supplementary Table S6). MiRNA167 can further influence the development of pollen mother cells to pollen grains by cutting the auxin response factors *LOC_Os10g33940, LOC_Os02g06910, LOC_Os04g57610, LOC_Os06g46410,* and *LOC_Os12g41950* [24]. MiRNA164e was used as an *OsDBH* (DEAD Box Helicase) gene to encode helicase under simulated salt stress, and it adapted to salt stress by regulating the expression of helicase-related genes [25]. The decreased expression level of miR398a and 398b under aluminum stress can cause the upregulation of the target genes *AtSOD1* and *AtSOD2* (superoxide dismutase1/2), while SOD is an important enzyme that promotes the conversion of superoxide free radicals into hydrogen peroxide and oxygen to reduce cell damage [26]. *Phyb, DBH, SOD1*, and *SOD2* were also dissimilated at different developmental stages and under two light treatments (Supplementary Table S6). In addition, many circRNAs in this study have not been discovered previously, which also indicates that more needs to be understood regarding the regulation of targeted miRNA by circRNA. The results showed that the functions of these known targeted miRNAs are not directly related to the grain development and filling processes. There are two main reasons for this. Firstly, during the development of grains under different light conditions, circRNA might be regulated by a sponge mechanism targeting miRNA. In previous studies, it has also been suggested that during plant growth, circRNA might also regulate the entire biological process by regulating the expression level of the parent gene [9]. Secondly, there is little evidence of miRNAs in plants playing a role in light regulation [27], and there are a large number of undiscovered miRNAs in this study. These unknown miRNAs might act on grain filling and be targeted by circRNAs.

Abscisic acid regulates sugar metabolism during stress, and α-amylase breaks down starch into fructose and glucose, which has a particular negative regulation effect on the decomposition of sucrose [28]. Sucrose enters the starch synthesis pathway after being transported to the rice grain through vascular bundles, and it regulates changes in sugar signals, inducing changes to various metabolic cycles in the grain. Studies have shown that sugar starvation may induce the expression of an amylase synthesis gene and improving its activity. Reductions in starch synthesis result in a decrease in starch accumulation and rice quality [15].

Abscisic acid reduces the activity of ATPase, reduces the transporting force of H+ across the plasma membrane, and then affects the H+/sucrose co-transport pathway [29]. During the grain-filling period, the lower ABA levels in the early stage and the higher ABA level in the middle stage were not conducive to rice grain filling. The application of exogenous ABA at an appropriate concentration in the early stage of grain filling can promote the development of grain embryos, assimilate transport, promote grain filling, and increase the seed setting rate and yield of rice [30,31]. Zhang et al. [32] used the method of tracer dynamics analysis to apply ABA to paddy rice ears. This can inhibit the formation of temporarily non-exportable substances in the flag leaves, promote the formation of transmissible substances, and increase the output rate of photosynthetic products. After this, it has an inhibitory effect on structural substances and respiratory consumption, and it has a promotion effect on the formation of injectable substances. Therefore, although photosynthesis was inhibited at the early stage of grain filling in this study, the endogenous ABA in the grains did not decrease, the sucrose content was still maintained at a certain level, and the dry weight of the entire ear was not significantly inhibited.

The ABA biosynthesis-related gene expression of the low light treatment was significantly different from the control group at 10 days after flowering. Abscisic acid can participate in a series of processes in the adversity stress reaction of plants. It can improve the free proline, soluble sugar, and sucrose osmotic regulation substances, as well as enhance the capacity of osmotic regulation. In this study, the ABA content increased at the same time as the sucrose content, indicating that grain under weak light can the exhibit regulation of endogenous ABA to the detriment of resistance to abiotic stress. When the drought stress was postponed to reduce the water content of sesame leaves, the content of MDA increased significantly, and the content of soluble sugar increased first and then decreased [33]. This result is consistent with our results (Figure 1).

Yang et al. [34] reported that the regulation effect of ABA on grain filling showed a dose effect: the concentration was promoted, but a high concentration was inhibited, as ethylene could reduce the critical enzyme activity of the sucrose–starch metabolism pathway in the grain and inhibit grain filling. The interaction of ABA and ethylene regulates grain filling. Therefore, appropriately increasing the ABA level and the ratio of ABA to ethylene using methods such as moderate soil drought can promote grain filling in rice and wheat.

In a study on wheat, 9–14 days of spraying low concentrations of exogenous ABA was found to improve wheat grain sucrose synthetase (SuSase), soluble starch synthase, and ADP glucose focal phosphorylase activity [35]. Among these, SuSase activity is closely related to library strength and is considered an indicator of library strength. This finding infers that ABA promotes grain filling by increasing the reservoir strength by regulating the critical enzyme activity of sucrose–starch metabolism in grains [36] Peng, H.

## 4. Materials and Methods

### 4.1. Plant Materials and Stress Treatments

Rice seeds, *Oryza sativa* Linnaeus, of the Shuhui 498 variety were placed in barrels that were 27 cm high and 33 cm in diameter, and whole plants were placed in a Conviron A1000PG artificial climate box (Winnipeg, Canada) in 2018 and 2019 with light intensity of 700 mol.m^−2^ s^−1^ for a photoperiod of 14 h at a temperature of 29 °C during the day and a temperature of 20 °C during the 10 h night period (humidity: 75%) when rice was at the heading stage. At the beginning of the flowering period, the rice plants of low light treatment were exposed to a light treatment of 233 mol·m^−2^ ·s^−1^. The glume was removed from the rice grains at the filling stage for RNA separation at 5, 10, and 15 days after treatment with low light in 2018 and 2019. The representative grains in the middle of the rice panicle except the superior ones were selected as samples. In the control group, except for the fact that the light was normal, the sample collection method was consistent with the treatment.

### 4.2. Total RNA Extraction, circRNA Library Construction, and Sequencing

Total RNA was extracted from the control group and low light stress rice seeds using TRIzol reagent (Invitrogen Corporation, Carlsbad, CA, USA). The total RNA from each sample was used to prepare the circRNA sequencing library. After the RNA samples were qualified for detection, the rRNA was removed from the total RNA sample using the ribo-zero ™ kit. Some long noncoding RNAs (lncRNAs) have the same polyA-tailed structure as mRNA, so the removal of rRNA can maximize the retention of lncRNAs containing polyA-tailed RNA. A fragmentation buffer was added to the enriched RNA to break the RNA into small fragments. Then, a library was constructed using the segmented RNA as a template, and Qubit 2.0 (USA) was used for initial quantification and dilution of the library. After this, Agilent 2100 (USA) was used to detect the inserted fragment size of the library. After it was established that the inserted fragment size met the expectations, real-time PCR was used to accurately quantify the effective concentration of the library to ensure quality of the library. After successfully passing library detection, different libraries were pooled into flow cells according to the requirements for effective concentration and target disembarkation data volume. After cBOT clustering, the Illumina HiSeqX high-throughput sequencing platform was used for sequencing.

### 4.3. Identification of Circular RNAs

The filtered transcriptome sequencing data from each sample were combined, and four software programs, Starchip v 1.3e, CIRI2 v 2.0, CIRCexplorer2 1.1.10, and CircRNA_finder 1.1, were independently used to predict the back-spliced junction. The predicted circRNA genome sequence (osa_predict_seq.fa, as the query sequence) was compared with the known circRNA database genome sequence or transcriptome sequence to complete the preliminary verification of the circRNA. The Plantcircbase database, for genome sequences, was used with the Blastn ratio and a filtering condition of subject coverage greater than 90%, and the Plantcircnet database, for transcriptome sequences, was also used to identify the circRNA that was in the intergenic region or single exon region (i.e., circRNA spans multiple exons) with filtering conditions of more than 90% similarity and lengths greater than 100.

### 4.4. Prediction of miRNA Targets of circRNAs, mRNA Targets of miRNA, and Annotation of Functions

The sequence in the middle of the circRNA’s trans-shear site was extracted, and the reverse sequence was spliced from the middle and extended by default by 15 nt. The circRNA–miRNA regulatory relationship was predicted using TargetFinder 1.7, using sequence-based and free-energy-based calculations. The TargetFinder 1.7 result filter parameter that was used was a score of three. Regulatory networks were constructed for the control group and low light treatment for the period of 5–10 days after flowering using differential expression (DE) circRNA–DE miRNA and DE miRNA–DE mRNA, and then enrichment analysis was conducted. The filtering conditions of DE circRNA–DE miRNA were predicted using at least one predictive software. The filtering condition for the pairs of DE miRNA–DE mRNA effects was that the *p*-value of the expression correlation coefficient was less than 0.05 and was predicted by at least one predictive software program (Supplemental Figures S1–S3). An analysis of targeted messenger RNA functions was performed using the Gene Ontology (GO), Kyoto Encyclopedia of Genes and Genomes (KEGG), and Wikipathway databases to predict the processes involved in rice grain development, such as photosynthesis, sugar and starch synthesis and metabolism, and the signaling of plant hormones, such as abscisic acid and auxin. Then, the functional annotation results of the GO, KEGG, and Wikipathway databases were used to analyze the enrichment of differentially expressed mRNAs in the network. Each sample was repeated three times.

### 4.5. Validation of Differentially Expressed Circular RNAs

The samples collected as described in Section 4.1 are used for the validation of the expressed circular RNAs. Each sample was repeated three times. Quantitative real-time PCR and Sanger sequencing techniques were used to verify the circular structure and expression patterns of circRNAs identified using RNA sequences. During validation, 15 differentially expressed circRNAs were used, of which 6 were predicted to be sponges for more than 2 miRNAs, and 9 were selected randomly from differentially expressed circRNAs. Two micrograms of total RNA was used before real-time quantitative PCR with DNase I (2270 a, Takara, Japan). Primers were designed to ensure that the circular template was amplified [37]. The sequence of primers is shown in Supplementary Table S1. The expression of circRNAs was quantified on an ABI StepOnePlus system (USA) using an SYBR green master mixture (Applied Biosystems, Foster City, CA, USA). The relative expression rate (∆∆Ct) of each circRNA was calculated using the 2^−^^∆∆Ct^ method and expressed as log_2_ of the value, where Ct is the periodic threshold value of the amplified target or reference gene [38]. The expression of *OsActin* was used as a reference for data normalization. SPSS Statistics 19.0 software was used for statistical analysis. The Student’s *t*-test was used to compare the significant difference between the control and the light treatment group using a probability level of 0.05.

### 4.6. Quantification of Sucrose and ABA (Abscisic Acid)

Approximately 100 mg of developing caryopses was used to quantify the ABA and sucrose content at the grain-filling stage 5, 10, and 15 days after treatment in both the light stress and the control groups, using liquid chromatography-tandem mass spectrometry as described previously [39]. A total of 0.2 g of the sample was accurately weighed, and 2 mL of 80% methanol aqueous solution containing 0.5% formic acid was pre-cooled at 4 °C as the extraction solution. After 30 min of extraction, the sample was still overnight. Supernatant was obtained by centrifugation at 12,000 r/min at 4 °C for 15 min. It was evaporated to the water phase by rotation in a water bath kept at a constant temperature of 38 °C constant temperature water bath and frozen for 30 min in a -20 °C refrigerator. The supernatant was centrifuged at 12,000 r/min at 4 °C for 10 min before being concentrated to near dry with a nitrogen blowing instrument, and then redissolved with 1.0 mL acetonitrile solution. The filtrate was detected by high-performance liquid chromatography (Agilent 1260, USA) after 30 s ultrasonic treatment through a 0.22 μm microporous membrane.

## 5. Conclusions

In this study, by concentrating the analyses of different databases, it was concluded that circRNA hormone synthesis had certain influencing factors, including helicase, sod-related enzyme, and ABA metabolism, which further affected the synthesis and metabolism of sucrose decomposable starch, and finally affected the filling and grain quality. In addition, there were significant differences in the number and expression of circRNA in rice grains at different growth stages, indicating that circRNA plays an important role in the development of rice grains.

## Figures and Tables

**Table 1:** CircRNA differentially expressed genes summary.

Number	CK-5d	CK-10d	CK-15d	S-5d	S-10d	S-15d
CK-5d	0	0	0	0	0	0
CK-10d	28	0	0	0	0	0
CK-15d	0	74	0	0	0	0
S-5d	0	0	0	0	0	0
S-10d	0	0	0	124	0	0
S-15d	0	0	0	0	1	0

## Data Availability

Not applicable.

## Supplementary Materials

The following supporting information can be downloaded at: https://www.mdpi.com/article/10.3390/plants11091272/s1, Figure S1: Comparison of GO, KEGG and Wikipathway enrichment analyses of differentially expressed circRNA targeting expressed miRNA in control grains at 5 days and 10 days after normal light. a. GO enrichment results. b. KEGG enrichment results. c. Wikipathway enrichment results. Figure S2: Comparison of GO, KEGG and Wikipathway enrichment analyses of differentially expressed circRNA targeting expressed miRNA in control grains at 5 days and 10 days after low light treatment. a. GO enrichment results. b. KEGG enrichment results. c. Wikipathway enrichment results. Figure S3: Comparison of GO, KEGG and Wikipathway enrichment analyses of differentially expressed circRNA targeting expressed miRNA in low light grains at 10 days and 15 days after normal light. a. GO enrichment results. b. KEGG enrichment results. c. Wikipathway enrichment results; Table S1: Primers in the quantitative RealTime-PCR assay for validating the expressions of circRNAs. Table S2: The candidate circRNAs that were verified using the Plantcircbase and Plantcircnet databases. Table S3: Differential expression of circRNAs between different light treatments and different development times. Table S4: The information of circRNA targeted miRNA. Table S5: The information of circleRNA-miRNA-mRNA which was significantly differentially expressed between 5 days and 10 days under low light condition. Table S6: Differences in miRNA and mRNA expression during grain filling in rice under low light. Table S7: Code list of Figure 3.
