# Supplementary figures and images for: Low Light Conditions Alter Genome-Wide Profiles of Circular RNAs in Rice Grains during Grain Filling
Source: Plants (Basel). 2022 May 9;11(9):1272. doi: 10.3390/plants11091272 (PMC9102277; doi:10.3390/plants11091272)

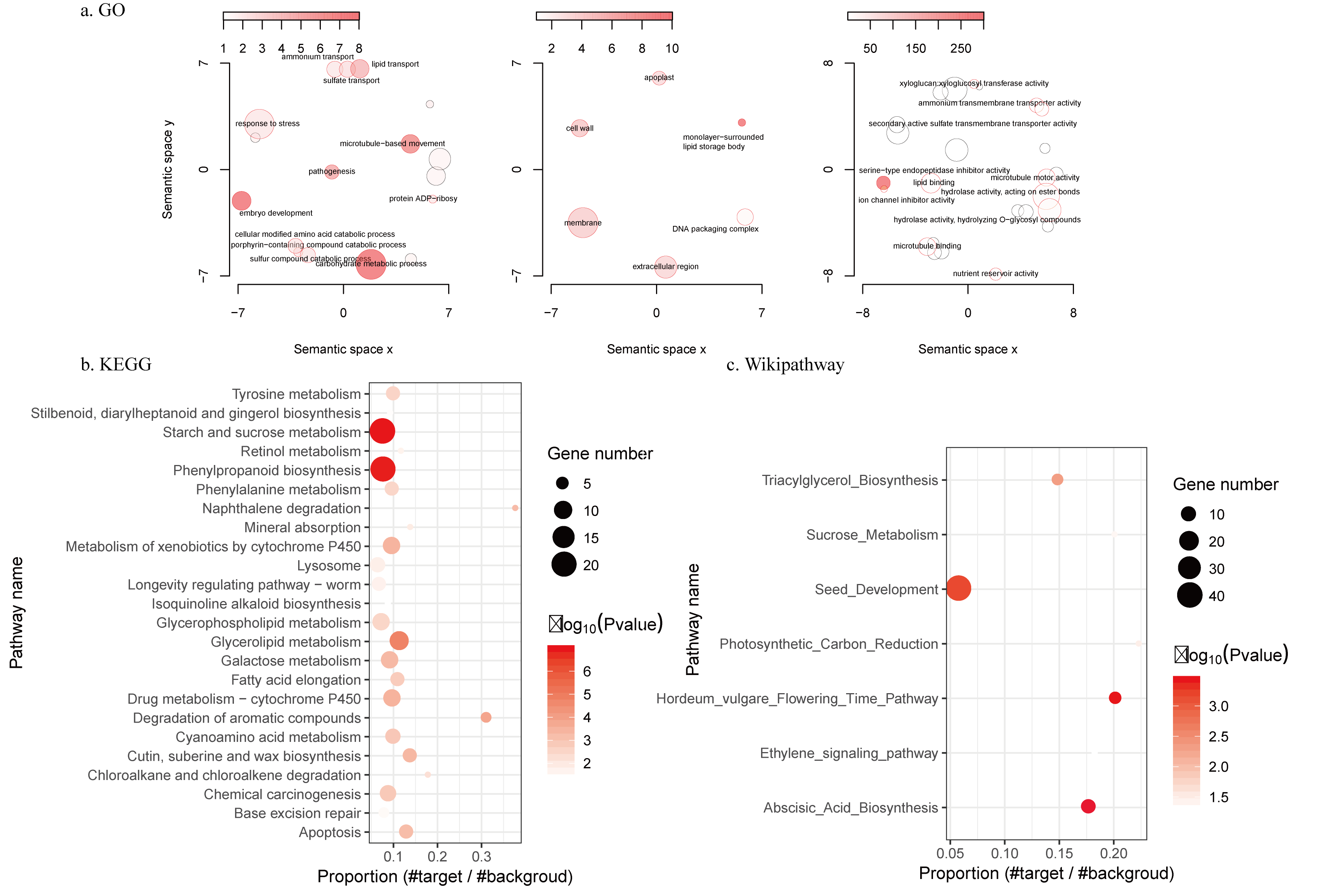

Supplement: Supplementary file 1 [file plants-11-01272-s001.zip › supplemental fig S1.tif]

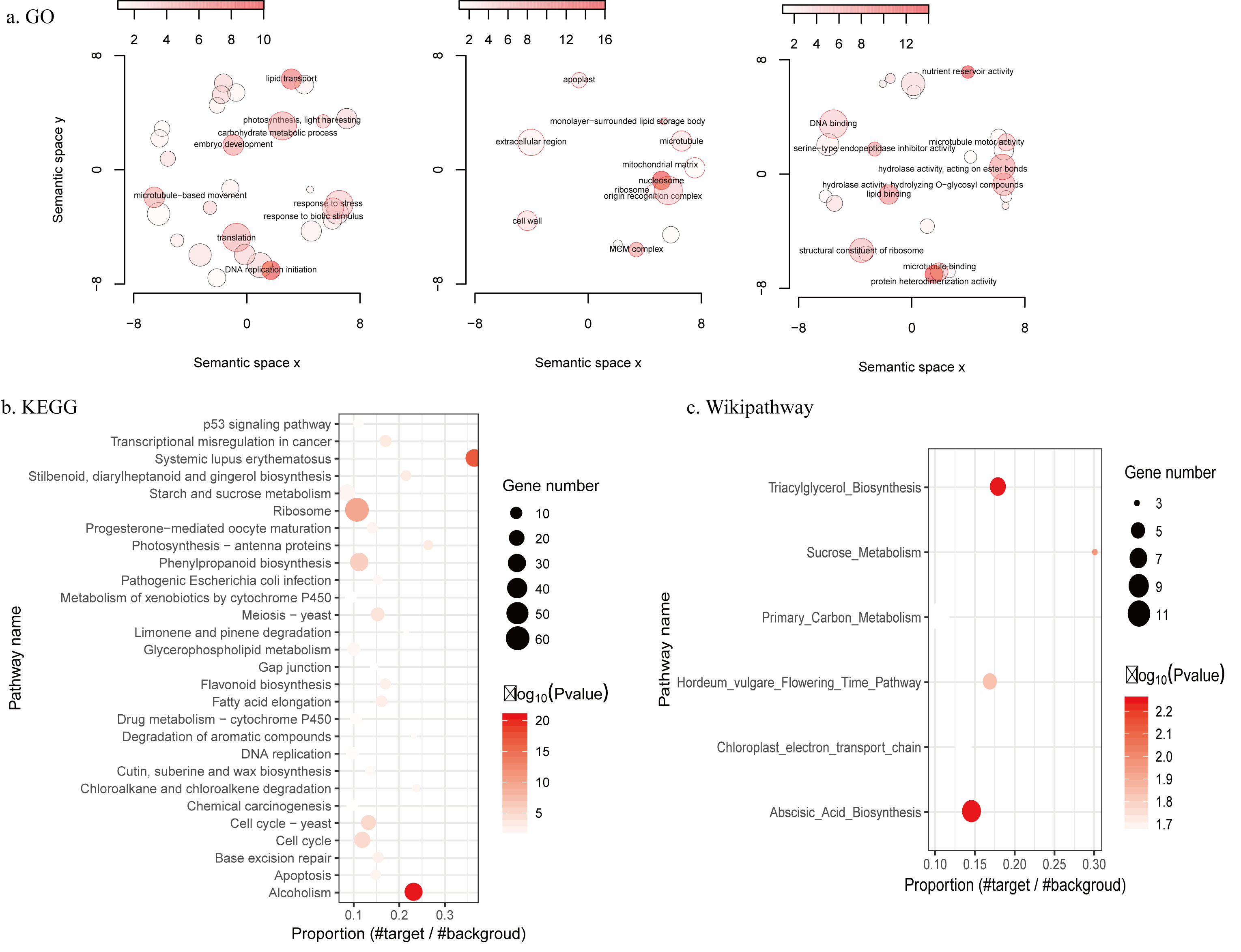

Supplement: Supplementary file 1 [file plants-11-01272-s001.zip › supplemental fig S2.tif]

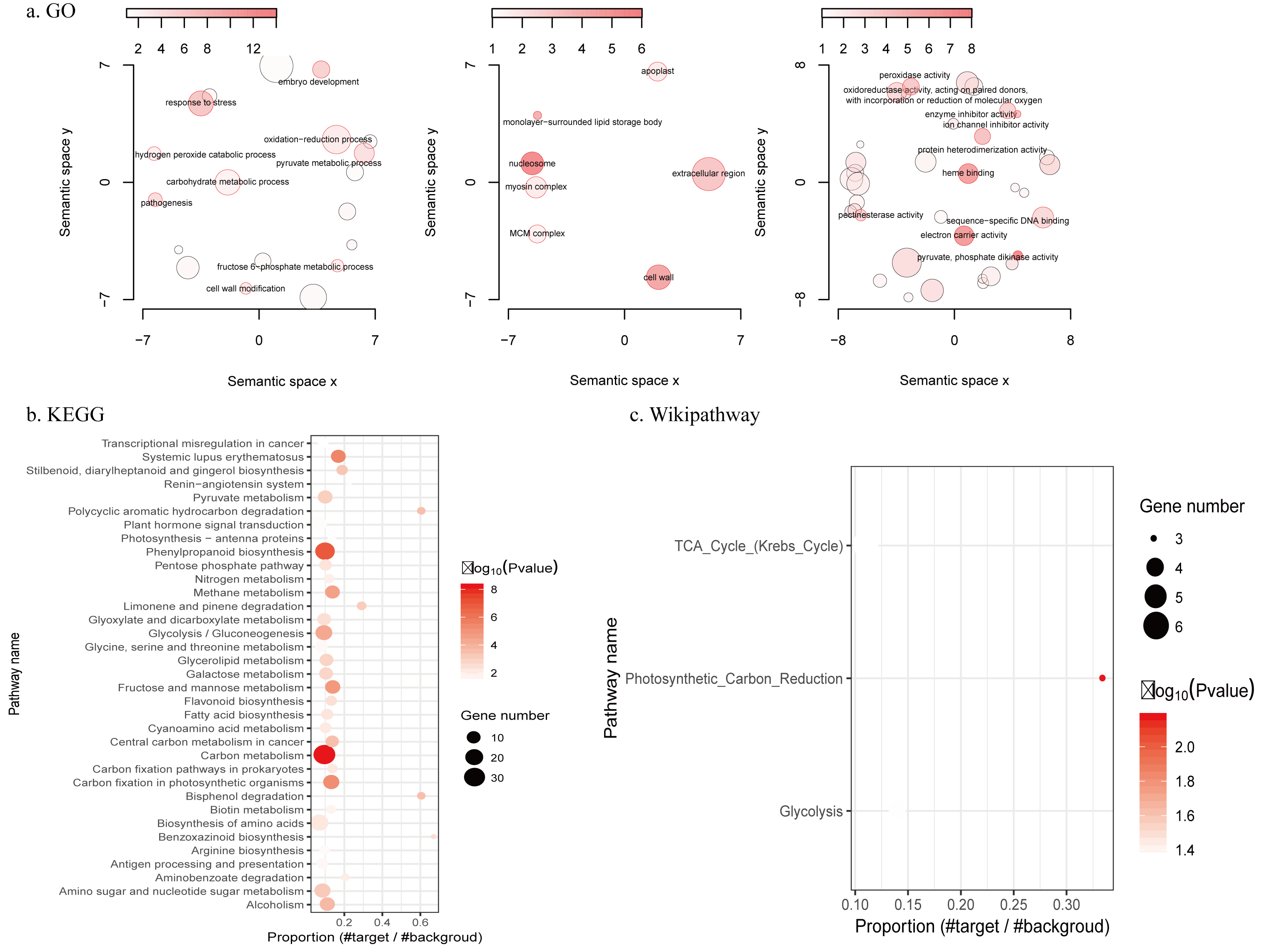

Supplement: Supplementary file 1 [file plants-11-01272-s001.zip › supplemental fig S3.tif]
